# The role of early functional neuroimaging in predicting neurodevelopmental outcomes in neonatal encephalopathy

**DOI:** 10.1007/s00431-022-04778-0

**Published:** 2023-01-06

**Authors:** Carla R. Pinto, João V. Duarte, Carla Marques, Inês N. Vicente, Catarina Paiva, João Éloi, Daniela J. Pereira, Bárbara R. Correia, Miguel Castelo-Branco, Guiomar Oliveira

**Affiliations:** 1 Pediatric Intensive Care Unit, Hospital Pediátrico, Centro Hospitalar e Universitário de Coimbra, Avenida Afonso Romão, Coimbra, 3000-602 Portugal; 2 University Clinic of Pediatrics, Faculty of Medicine, University of Coimbra, Coimbra, Portugal; 3 Coimbra Institute for Biomedical Imaging and Translational Research (CIBIT), Institute of Nuclear Sciences Applied to Health (ICNAS), University of Coimbra, Coimbra, Portugal; 4 Faculty of Medicine, University of Coimbra, Coimbra, Portugal; 5 Child Developmental Center, Research and Clinical Training Center, Hospital Pediátrico, Centro Hospitalar e Universitário de Coimbra, Coimbra, Portugal; 6 Ophthalmology Department, Centro Hospitalar e Universitário de Coimbra, Coimbra, Portugal; 7 Otorhinolaryngology Department, Centro Hospitalar e Universitário de Coimbra, Coimbra, Portugal; 8 Neuroradiology Unit, Medical Imaging Department, Centro Hospitalar e Universitário de Coimbra, Coimbra, Portugal; 9 Quantitative Methods, Information and Management Systems Department, Coimbra Business School, Coimbra, Portugal

**Keywords:** Perinatal asphyxia, Neonatal encephalopathy, Newborn, Prognosis, Functional neuroimaging, Neurodevelopmental outcome

## Abstract

Reliably assessing the early neurodevelopmental outcomes in infants with neonatal encephalopathy (NE) is of utmost importance to advise parents and implement early and personalized interventions. We aimed to evaluate the accuracy of neuroimaging modalities, including functional magnetic resonance imaging (fMRI) in predicting neurodevelopmental outcomes in NE. Eighteen newborns with NE due to presumed perinatal asphyxia (PA) were included in the study, 16 of whom underwent therapeutic hypothermia. Structural magnetic resonance imaging (MRI), and fMRI during passive visual, auditory, and sensorimotor stimulation were acquired between the 10th and 14th day of age. Clinical follow-up protocol included visual and auditory evoked potentials and a detailed neurodevelopmental evaluation at 12 and 18 months of age. Infants were divided according to sensory and neurodevelopmental outcome: severe, moderate disability, or normal. Structural MRI findings were the best predictor of severe disability with an AUC close to 1.0. There were no good predictors to discriminate between moderate disability versus normal outcome. Nevertheless, structural MRI measures showed a significant correlation with the scores of neurodevelopmental assessments. During sensorimotor stimulation, the fMRI signal in the right hemisphere had an AUC of 0.9 to predict absence of cerebral palsy (CP). fMRI measures during auditory and visual stimulation did not predict sensorineural hearing loss or cerebral visual impairment.

*Conclusion*: In addition to structural MRI, fMRI with sensorimotor stimulation may open the gate to improve the knowledge of neurodevelopmental/motor prognosis if proven in a larger cohort of newborns with NE.

**Table:** 

**What is Known:**
• *Establishing an early, accurate neurodevelopmental prognosis in neonatal encephalopathy remains challenging.*
• *Although structural MRI has a central role in neonatal encephalopathy, advanced MRI modalities are gradually being explored to optimize neurodevelopmental outcome knowledge.*
**What is New:**
• *Newborns who later developed cerebral palsy had a trend towards lower fMRI measures in the right sensorimotor area during sensorimotor stimulation.*
• *These preliminary fMRI results may improve future early delineation of motor prognosis in neonatal encephalopathy.*

**Supplementary Information:**

The online version contains supplementary material available at 10.1007/s00431-022-04778-0.

## Introduction

Neonatal encephalopathy (NE) succeeding perinatal asphyxia (PA) contributes considerably to poor neurodevelopmental outcomes [1]. Possible long-term neurodevelopmental sequelae, among survivors, include cerebral palsy (CP), intellectual disabilities, epilepsy, vision, and hearing impairments. Despite early therapeutic hypothermia (TH), current results in high-income countries reveal that the CP rate has remained unchanged around 20% [1; 2], while hearing and visual impairments occur less frequently [2]. It is well recognized that neonates with severe NE at birth have a higher probability of death or disabling neurological and cognitive deficits. However, the prognosis of newborns with mild and moderate NE is more variable, with milder motor deficits and a broader spectrum of cognitive impairments, making prediction more difficult [1, 3]. Therefore, establishing an accurate early neurodevelopmental prognosis in newborns with brain injury, especially in those who will develop a moderate disability, remains a challenging task in the neonatal intensive care unit. Although structural magnetic resonance imaging (MRI) is a robust predictor of neurodevelopmental outcome in newborns with NE due to presumed PA, irrespective of TH [4], it has recently been demonstrated that spectroscopy may have a better accuracy, but requires expertise and harmonization [5]. Hence, currently structural MRI remains the most widely used technique, as it allows to characterize the degree and pattern of brain lesions. However, it is often insufficient to predict long-term disabilities such as fine motor, social, behavioral, sensorial, and cognitive deficits, in particular in mild to moderate cases of NE [6]. Thus, new methods to assess brain function early to optimize prognostic information are lacking [7]. Functional magnetic resonance imaging (fMRI), better established in adults that also requires expertise, could potentially be considered the technique of choice for functional assessment of the newborn brain [7]. We hypothesize that fMRI might identify early brain function changes in neonates with NE related to neurodevelopmental impairments before clinical evidence of pathological signs. If this potential holds true, it could be of extreme importance, given that neuroplasticity and brain adaptability are well documented as most modifiable in neonates [8]. Identifying, as early as possible, functionally affected systems offer considerable likelihood for optimizing brain outcomes by implementing intensive and targeted psychoeducational and therapeutic interventions. The main objective of this study was to evaluate the accuracy of neuroimaging modalities, including fMRI, to predict severe or moderate disability at 18 months of age in newborns with NE due to presumed PA. Additionally, we intend to specifically relate blood-oxygen-level-dependent (BOLD) signal changes, measured with fMRI, during visual, auditory, and sensorimotor stimulation, with detailed sensory and neurodevelopmental function at 12 and 18 months of age, respectively.

## Materials and methods

### Participants

An observational, exploratory study with prospective data collection was conducted. All procedures have been carried out in accordance with the Code of Ethics of the World Medical Association (Declaration of Helsinki) for experiments involving humans. Informed written consent was obtained from parents of all participants after a full verbal and written explanation of the study. The study was approved by the Ethics Committee of the Faculdade de Medicina da Universidade de Coimbra, Portugal (Reference CE-029–2014). All newborns admitted to the pediatric intensive care unit (PICU) of a tertiary pediatric hospital from Centro Hospitalar e Universitário de Coimbra, Portugal, born at or after 36 gestational weeks, with NE due to presumed PA, defined according to the American College of Obstetricians and Gynecologists’ Task Force on Neonatal Encephalopathy, 2014 criteria [9], were recruited between April 2016 and March 2017. Exclusion criteria were presence of major congenital abnormalities and inherited inborn errors of metabolism or stroke. Neonatal data collection was retrieved from the PICU electronic clinical database and comprised parents and neonatal characteristics, assessment, evolution, and treatment. Socio-economic status of parents was categorized according to the International Standard Classification of Education (ISCED) and the Portuguese version for European Deprivation Index (EDI-PT) [10]. The NE was characterized with the Modified Sarnat and Thompson scores, applied on admission [11, 12]. The amplitude-integrated electroencephalography (aEEG) worst background pattern at admission and 48 to 72 h of age was classified according to Hellstrom-Westas, 2006 [13].

### MRI data acquisition

MRI data were acquired at a mean age of 12 ± 3 days of age, using a 3 T scanner (Siemens AG, Healthcare, Erlangen, Germany), with a 20-channel head coil. Foam cushions and sedation with intravenous midazolam or propofol were used to minimize head motion. The MRI protocol included a 3D T1-weighted MPRAGE (0.83-mm isotropic voxel, 160 slices, TR/TE = 2300/3.5 ms), a 3D T2-weighted SPACE (0.83-mm isotropic voxel, 160 slices, TR/TE = 3200/443 ms), diffusion-weighted imaging (DWI), and three fMRI T2*-weighted sequences sensitive to BOLD (blood-level-oxygen-dependent) contrast (TR/TE = 2080/31 ms, voxel size = 2 × 2 × 2 mm^3^, 29 axial slices (whole-brain coverage), FOV = 256 × 256 mm^2^, FA = 90°). Continuous monitoring of the newborns inside the scanner was provided by an intensive care pediatrician and nurse.

### Structural MRI grading system of brain injury

Brain lesions characteristic of NE due to presumed PA were graded by two expert neuroradiologists using T1, T2, and DWI sequences, according to a recently reported scoring system [14].

### Functional stimulation paradigm

Newborns were submitted to passive visual [7], auditory [15], and sensorimotor stimulation [16], delivered in an optimized block design paradigm [17] in separate runs, described in detail in Online Resource 1.

### fMRI data preprocessing

Data were processed using BrainVoyager version 21.2 (Brain Innovation, Maastricht, The Netherlands), and the FMRIB Software Library (FSL) version 4.1.8. fMRI volumes were corrected for motion using rigid transformations and motion parameters were included in data analysis [18]. Motion outliers were accounted through scrubbing of pairs of volumes with > 0.5 mm of translation or 0.5° of rotation between them. We applied slice-scanning time correction, linear trends removal, and temporal high-pass filtering (5 cycles per run). We then performed anatomical-functional registration and finally applied slight spatial smoothing (full-width half-maximum kernel with 3 mm) to functional data.

### fMRI subject-level data analysis

A whole-brain voxel-wise general linear model (GLM) was used to estimate the BOLD response to visual, auditory, or sensorimotor stimulation, using a two-gamma hemodynamic response function adapted for term infants [19]. While using a less conservative threshold of *p* < 0.05, as previously suggested for newborn fMRI studies [7], to compensate for multiple comparisons, only BOLD signal changes in voxels located in primary visual, auditory, or sensorimotor areas identified by visual inspection were considered, and regions of interest (ROIs) were defined as the set of activated voxels within these local anatomical sensitive search spaces.

### Sensory assessment at 12 months

The formal auditory and visual assessment included brainstem auditory and visual evoked potentials, described in Online Resource 2 and 3.

### Neurodevelopmental assessment at 18 months

Neurodevelopmental and clinical assessment was performed at a mean age of 19 ± 3 months by an experienced neurodevelopment team (neurodevelopmental pediatrician and a psychologist). Besides a detailed classical clinical (including growth evaluation) and neurological examination, these children were submitted to the following assessment tools, currently used in the neurodevelopment unity: the Griffiths Mental Development Scales (GMDS) [20], the Vineland Adaptive Behavior Scales (VABS) [21], and the Modified Checklist for Autism in Toddlers (M-CHAT) [22] for autism spectrum disorders (ASD) screening. Children with a positive M-CHAT, and according to clinical judgment, performed a direct structured proband instrument observation, the Autism Diagnostic Observation Schedule (ADOS), to confirm ASD suspected diagnosis [23]. CP was diagnosed using the clinical criteria from the European Network of Cerebral Palsy [24]. The Gross Motor Function Classification System (GMFCS) for CP classification was also applied. The neurological examination performed was standardized according to the Hammersmith Infant Neurological Examination (HINE) [25]. Epilepsy was diagnosed using the International League Against Epilepsy clinical definition [26]. Microcephaly was defined as head circumference more than two standard deviations (SD) below the mean for age and sex (i.e., less than the 3rd percentile) in the WHO growth charts.

### Outcomes at 18 months

Neurodevelopmental outcomes at 18 months of age were classified into three categories:

- Severe disability, defined if one of the following: (1) death; (2) global developmental quotient (DQ) in GMDS or VABS (total score, adaptive behavior composite - ABC), of 70 or below; (3) CP with a GMFCS 3–5; (4) cerebral visual impairment; (5) sensorineural hearing loss requiring amplification; (6) epilepsy (requiring anticonvulsant therapy at time of assessment); (7) confirmed ASD diagnosis.
- Moderate disability, defined if one of the following: (1) GMDS (global DQ) or VABS (total score, ABC) between 70 and 84; (2) CP with GMF 1–2.
- Normal, if GMDS (global DQ) or VABS (total score, ABC) above 84 and no clinical neurological, or neurodevelopmental sequelae at the time of assessment.

### Statistical analysis

The IBM-SPSS^®^ software version 27 was used. A level of statistical significance of 5% was considered. A univariate analysis was made, in which central tendency and dispersion measures were calculated for quantitative variables, and absolute and relative frequencies were determined for qualitative variables. Quantitative variables were first checked for normality using the Kolmogorov–Smirnov tests. Receiver operating characteristic (ROC) curves were performed to evaluate the accuracy of neuroimaging biomarkers to predict outcome. The power of the predictors to discriminate was quantified by the area under the ROC curve (AUC). Independent Student’s *t* test or Mann–Whitney *U* test were performed as appropriate to compare quantitative variables. Additionally, Spearman correlations were used to evaluate the relationship between quantitative variables without normal distribution.

## Results

During the study period, 20 newborns were eligible, of whom two were excluded: one died on the fifth day of age due to an adverse prognosis and redirecting of care and, and the other, had an inherited inborn error of metabolism. The demographic and clinical characteristics of the 18 newborns and their parents are described in Table 1. Parameters regarding PICU assessment, evolution, and treatment are summarized in Table 2. Sixteen newborns were submitted to TH. Regarding structural MRI, the median of the total score was 2 (IQR 1 to 6.75), the median of deep grey matter subscore was 0 (IQR 0 to 1.5), and the median of white matter/cortex subscore was 0 (IQR 0 to 5.25). No lesions in the cerebellum were observed. Three newborns had a total score of 0, four newborns had a total score of 1, scored in the additional subscore due to subdural hemorrhages, and in four newborns the total score ranged from 15 to 26, all of whom had lesions in the thalamus/basal ganglia, posterior limb of the internal capsule, or perirolandic cortex. Regarding fMRI, the summary measures of brain responses during visual, auditory, and sensorimotor stimulation from each newborn can be observed in Online Resources 4. No differences were observed in fMRI BOLD responses in newborns sedated with midazolam or propofol (Online Resources 5). Data from clinical and neurodevelopmental evaluation are summarized in Table 3 and in Online Resources 6. The median of HINE at 18 months of age was 78 (IQR 70 to 78) and five had a suboptimal score (< 74), four of whom had the highest punctuation in MRI deep gray matter subscore (6 to 15). Concerning children with positive score for ASD screening, M-CHAT (*n* = 5), four had CP or severe global psychomotor delay (GMDS global DQ or VABS total score < 70), and in one child ADOS was conducted, which ruled out ASD.

**Table 1:** Demographic and clinical characteristics of newborns with neonatal encephalopathy and their parents

**Demographic and clinical characteristics**	**NE** (*n* = 18)
**Age at delivery**, years, mean ± SD	
Mothers	31.6 ± 6.5
Fathers	34.4 ± 8.6
**ISCED**, level, median (IQR)	
Mothers	5 (2 to 6)
Fathers	3 (2 to 6)
**Deprived families**	3
**Gestational age**, weeks, median (IQR)	39.5 (37.75 to 40.0)
**Birth weight**, g, mean ± SD	3,208 ± 396
**Gestational age-birth weight**	
Small for gestational age	2
Appropriated for gestational age	15
Large for gestational age	1
**Gender**	
Male/female	14/4
**Type of delivery**	
Vaginal	1
Vacuum extraction	5
Forceps	2
C-section	10
**Delivery complications**	
Uterine rupture	1
Cord prolapse	0
Shoulder dystocia	1
Obstructed labor	1
**Resuscitation needs**	
Positive ventilation pressure	4
Tracheal intubation	14
Chest compressions	6
Adrenaline	5
**Apgar score 5′**, median (IQR)	6 (5 to 6)
**Apgar score 10′**, median (IQR)	7 (6 to 8)
**pH** 1st hour of age, mean ± SD	7.00 ± 0.1
**Base excess** 1st hour of age, mean ± SD	-17.9 ± 4.3
**Lactate** on admission, mmol/L, mean ± SD	15.7 ± 4.6
**Grade of NE** on admission	
Mild	2
Moderate	11
Severe	5
**Thompson score** on admission, median (IQR)	10 (8 to 12)
**aEEG background pattern** on admission	
Continuous	2
Discontinuous	9
Burst suppression	3
Low voltage	4
Inactive, flat	0

*NE* neonatal encephalopathy, *ISCED* International Standard Classification of Education, *n* total number, *IQR* interquartile range, *SD* standard deviation, *aEEG* amplitude-integrated electroencephalography

**Table 2:** PICU assessment, evolution, and treatment

	**NE** (*n* = 18)
**aEEG background pattern** at 48–72 h of age,	
Continuous	5
Discontinuous	10
Burst suppression	1
Low voltage	2
Inactive, flat	0
**Seizures**^a^	6
**Therapeutic hypothermia**	16
**Sedation**	17
Morphine	16
Midazolam	4
Duration, h, median (IQR)	84 (70 to 91)
**Mechanical invasive ventilation**	17
Duration, h, median (IQR)	114 (104 to 147)
**Cardiovascular support**	15
Dopamine	15
Adrenaline	3
**Anticonvulsants**	7
Phenobarbital	7
Midazolam	3
Phenytoin	1
**Length of PICU stay**, days, mean ± SD	12.1 ± 6
**Mortality**	0

*aEEG* amplitude-integrated electroencephalography (background pattern), *h* hours, *IQR* interquartile range, *n* total number, *NE* neonatal encephalopathy, *PICU* pediatric intensive care unit, *SD* standard deviation

^a^Seizures assessed during the first 72 h of age

**Table 3:** Clinical and neurodevelopment evaluation at 18 months

	**NE** (*n* = 18)
**Outcome**	
Severe disability	4
Moderate disability	8
Normal	6
**Cerebral** **palsy**	4
Spastic	4
Bilateral	3
Unilateral	1
GMFCS 1–2	1
GMFCS 3–5	3
**HINE**, median (IQR)	78 (70 to 78)
**Growth**	
Weight, z score, mean ± SD	−0.342 ± 0.97
Length/height, z score, mean ± SD	− 0.693 ± 1.16
Microcephaly	4
**GMDS**, global DQ,	
< 70	3
70–84	1
≥ 85	14
**VABS**, total score-ABC	
< 70	4
70–84	8
≥ 85	6
**ASD**	
Screening, M-CHAT, positive	5
Confirmed	0
**Auditory evoked potentials**	
Sensorineural hearing loss requiring amplification (moderate)	1
**Visual evoked potentials**	
Cerebral visual impairment	2
**Epilepsy**	3
Requiring one anticonvulsant	2
Requiring two or more anticonvulsants	1
**Feeding difficulties**	1
Tube feeding	1
Gastrostomy	0

*ABC* adaptive behavior composite, *ASD* autism spectrum disorder, *DQ* development quotient, *GMDS* Griffiths Mental Development Scales, *GMFCS* Gross Motor Function Classification System, *HINE* Hammersmith Infant Neurological Examination, *IQR* interquartile range, *M-CHAT* Modified Checklist for Autism in Toddlers, *n* total number, *NE* neonatal encephalopathy, *VABS* Vineland Adaptive Behavior Scales

In Table 4, the accuracy of neuroimaging modalities to predict moderate and/or severe disability at 18 months can be acknowledged after application of ROC curves. Overall, the fMRI measurements were not good predictors of outcome. Nevertheless, the AUC of BOLD signal (% signal change) in the right hemisphere during sensorimotor stimulation was 0.9 (95% CI: 0.694–1.0; *p* = 0.086) to predict absence of CP (Online Resources 7). The mean of BOLD (% signal change) in the right hemisphere during sensorimotor stimulation was − 0.1275 and 0.7819 in patients with and without CP, respectively (*t* = 1.6; *p* = 0.141; Cohen’s *d* effect size = 1.239).

**Table 4:** Accuracy of the neuroimaging modalities to predict severe or moderate disability

	**Severe**	**Moderate**	**Moderate and severe**
	AUC [95% CI]	AUC [95% CI]	AUC [95% CI]
**Structural MRI**			
** Total score**	1.0 [1.0; 1.0]*	0.708 [0.431; 0.986]	0.806 [0.604; 1.0]*
** Deep grey matter subscore**	1.0 [1.0; 1.0]*	0.5 [0.184; 0.816]	0.667 [0.415; 0.918]
** White matter/cortex subscore**	1.0 [1.0; 1.0]*	0.625 [0.327; 0.923]	0.750 [0.526; 0.974]
**fMRI** BOLD mean (% signal change)			
** Stimulus** per brain hemisphere			
** Visual** left	0.611 [1.16; 1.0]		
** Visual** right	0.519 [0.103; 0.934]		
** Auditory** left	0.167 [0.0; 0.409]		
** Auditory** right	0.333 [0.0; 0.682]		
** Sensorimotor** left	0.667 [0.289; 1.0]		
** Sensorimotor** right	0.1 [0.0; 0.306]		

Severe vs moderate disability and normal outcome (*n* = 18); moderate disability vs normal (*n* = 14); moderate and severe disability vs normal (*n* = 18)

*AUC* area under the curve, *CI* confidence intervals, *h* hours, *MRI* magnetic resonance imaging, *fMRI* functional magnetic resonance imaging, *BOLD* blood-oxygen-level-dependent

^*^p < 0.05

**Fig. 1:**
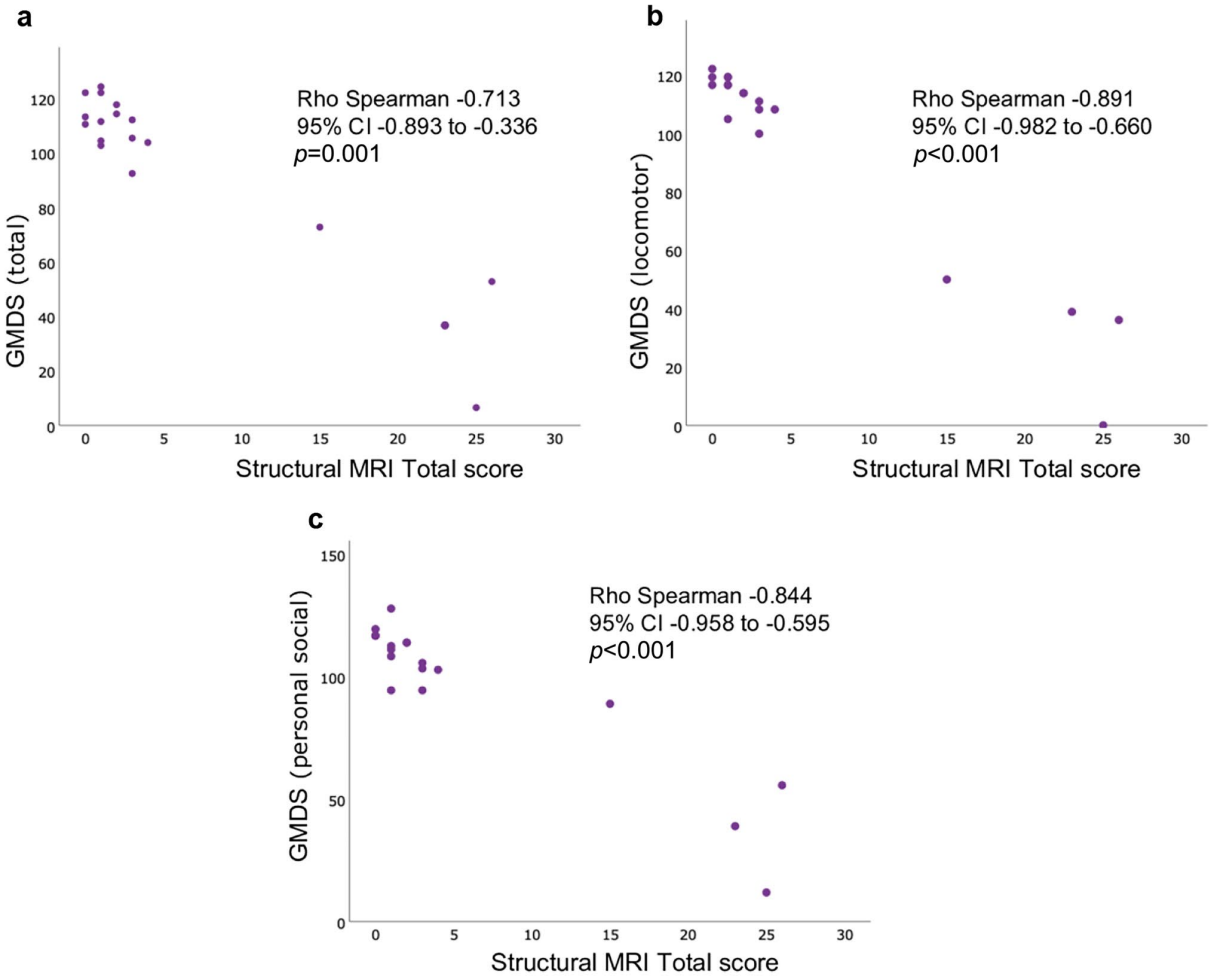
Correlation between GMDS and structural MRI score. Spearman correlation between the global developmental quotient (**a**), sub-quotient of the locomotor subscale (**b**), sub-quotient of the personal-social subscale (**c**) from GMDS and the total score of structural MRI. Global developmental quotient and sub-quotients are standardized to a mean of 100 and a standard deviation of 15. The higher the value, the higher mental development. The total score of structural MRI ranges from 0 to 55 points. The total score has a predictive value for outcome at 2 years and school age. The higher the score, the worse the prognosis. CI, confidence intervals; GMDS, Griffiths Mental Development Scales; MRI, magnetic resonance imaging

The significant relationships between the scores GMDS (total or subscales) and the structural MRI score can be observed in Fig. 1. Additionally, a positive Spearman correlation was found between the right brain hemisphere fMRI BOLD signal during sensorimotor stimulation and the GMDS locomotor subscale (Rho = 0.784; *p* = 0.003; 95% CI 0.413 to 0.956) or the HINE (Rho = 0.652; *p* = 0.022; 95% CI 0.262 to 0.890) at 18 months.

## Discussion

In our study, the best early predictor of severe disability at 18 months of age, with excellent performance (AUC near 1.0), was structural MRI, measured with the application of the Weeke score for classification of HIE brain lesions, despite performed near the time of DWI pseudonormalization. Its accuracy in predicting jointly moderate and severe neurodevelopmental disabilities relative to normal outcomes was lower, however reaching an AUC of 0.806. The structural MRI score also showed a high negative correlation (Rho Spearman from − 0.713 to − 0.891) with global DQ of GMDS and the sub-quotients from its locomotor and personal-social domains (Fig. 1). Although these results should be interpreted with caution, given the characteristics and small sample size, it is similar to that reported by Weeke et al. in one of the studied cohorts (AUC 0.988 for gray matter subscore) [14]. Different structural MRI scores to classify brain NE lesions due to presumed PA were proved to be highly predictive of outcomes at 24 months [27]. Some of those grading systems are based on categorizing defined brain injury patterns, so they are easy to apply. However, it is challenging to classify brain lesions in infants when they are not adequately included in the standard NE patterns. Accordingly, a current study exposes the limitations of the qualitative interpretation of structural MRI patterns [28]. The recently developed Weeke score [14] we used assesses all relevant brain areas separately and applies an item-based system; it can improve the detection of slight injury in mild NE [29] and has better accuracy and comparable inter-rater reliability relative to other item-based scoring systems [27].

Although we were unable to identify biomarkers that distinguish moderate neurodevelopmental disability from normal outcomes at 18 months of age, which is challenging, we observed a negative correlation between structural MRI total score and the results of neurodevelopmental assessment instruments that can improve the knowledge of the wide prognosis range in newborns with NE.

One of our main and innovative goals was to determine if fMRI can improve the capacity of structural MRI to predict neurodevelopmental outcomes, including potential damage to motor, visual, or auditory pathways. It is well recognized that structural MRI is a good predictor of severe CP, namely if severe basal ganglia/thalamus lesions or a clear abnormal posterior limb of the internal capsule signal are observed [30]. Nevertheless, it may not be as accurate in mild cases of CP [31]. We observed that fMRI measures (mean of BOLD % signal change) in the right brain hemisphere during sensorimotor stimulation were good predictors of CP absence. Children with CP had a trend for lower mean values of BOLD signal when compared to others, although without reaching statistical significance, probably due to the small sample size. Additionally, a positive significant correlation was established between those fMRI measures and the HINE or locomotor subscale of GMDS at 18 months of age. If replicated in larger-scale studies, these results may improve the ability to predict CP in newborns with NE before clinical evidence and promote early targeted educational interventions. We hypothesized that the negative responses observed in the right sensorimotor area of the newborns who later develop CP might be related to oxygen consumption associated with possible synaptogenesis due to brain plasticity and motor reorganization [8, 32]. These mechanisms might also explain why this relationship was only observed in the right hemisphere, in addition to the eventual maturational asymmetry, cortical hemisphere preference, or influence of the stimulus type.

Although the literature on fMRI in newborns with brain injury is still scarce, these findings are supported by a study performed on newborns with perinatal brain lesions, including NE due to presumed PA, which concluded that the CP group had reduced functional connectivity from the right supplementary motor areas when compared with the non-CP group [33]. However, they did not find any difference between the groups in brain activation during a motor task like the one used in our study.

We could not observe differences in standard brain responses during visual and auditory stimulation in newborns with cerebral visual impairment and sensorineural hearing loss when compared with others. These results can be explained due to the inconsistency of reports observed in previous fMRI studies in newborns, the difficulty of normalizing functional activity data or eventually being influenced by sedation used to avoid head motion. It is well recognized that neonatal fMRI studies observed adult-like positive BOLD responses, but also temporally delayed peak BOLD responses or smaller and negative responses [7, 19, 34, 35]. Possible explanations for this variability are the maturity level of neurovascular coupling and of autoregulation mechanisms and different cerebral oxygen metabolic rates in newborns [36]. Despite our efforts to analyze fMRI data with an age-appropriate hemodynamic response function, the best way to detect BOLD signals using task-based fMRI in newborns is not yet fully understood [19]. Furthermore, when using fMRI in newborns with brain injury, specific methodological aspects become more significant and should be considered [37]. Another contributing factor for the lack of power of fMRI analysis may be the predominant involvement of the white matter and thalamus, more than the cortex itself, in the hypoxic-ischemic perinatal insult, highly correlated with neurodevelopmental impairment, especially with motor and visual function [38–40]. Thus, normal primary visual cortex or normal optic radiations do not rule out the possibility of an abnormal visual function [40]. Conversely, reduced activation in the occipital cortex and functional connectivity in fMRI during visual stimulation was found in infants with perinatal brain injury [41].

The limitations of this study include a small sample of newborns with NE and a small number of those with CP, cerebral visual impairment, and sensorineural hearing loss, restricting the inference and generalization of results. The maturity level of the local neurovascular coupling underlying the BOLD signal can be potentially compromised in this population of high-risk newborns, precluding a good model of the hemodynamic response. Using a bilateral task for sensorimotor stimulation, instead of an alternating unilateral one, prevents us from thoroughly assessing lateralization and eventually achieving a better comprehension of motor injury and its reorganization. Additionally, the effects of the sedation used to avoid head motion on brain function cannot be entirely excluded.

However, this study has a number of strengths: use of a structured item-based system for classifying brain injury due to presumed PA; a comprehensive and detailed assessment of the neurodevelopmental outcome measures and infant’s needs, optimized with the use of complementary instruments, including VABS, and the multidisciplinary approach comprising otorhinolaryngology and ophthalmology evaluation; inclusion of the challenging fMRI application as a potential tool to improve motor injury prediction in the future.

## Conclusion

This exploratory and challenging outcome study of newborns with NE due to presumed PA strengthens structural MRI’s relevance to predict neurodevelopmental impairment. The still unmet possibility of fMRI providing a wealth of new information about the integration of functional cerebral activity in NE can be achieved through better defining normal inter-individual variability and tailored hemodynamic response function. Nevertheless, our fMRI results during sensorimotor stimulation open the door to refining future motor function characterization, allowing accurate prognostic information and early specific and precise therapeutic rehabilitation interventions.

## Supplementary Information

Below is the link to the electronic supplementary material.

Supplementary file1 (DOCX 20 KB)

Supplementary file2 (DOCX 17 KB)

Supplementary file3 (DOCX 19 KB)

Supplementary file4 (DOCX 23 KB)

Supplementary file5 (DOCX 18 KB)

Supplementary file6 (DOCX 19 KB)

Supplementary file7 (DOCX 18 KB)

## Abbreviations

ABC: Adaptive Behavior Composite score
ADOS: Autism Diagnostic Observation Schedule
aEEG: Amplitude-integrated electroencephalography
ASD: Autism spectrum disorder
AUC: Area under the curve
BOLD: Blood-oxygen-level-dependent
CI: Confidence interval
CP: Cerebral palsy
DQ: Developmental quotient
DWI: Diffusion-weighted imaging
EDI-PT: Portuguese version for European Deprivation Index
fMRI: Functional magnetic resonance imaging
GLM: General linear model
GMDS: Griffiths Mental Development Scales
GMFCS: Gross Motor Function Classification System
HIE: Hypoxic-ischemic encephalopathy
HINE: Hammersmith Infant Neurological Examination
IQR: Interquartile range
ISCED: International Standard Classification of Education
M-CHAT: Modified Checklist for Autism in Toddlers
MRI: Magnetic resonance imaging
NE: Neonatal encephalopathy
PA: Perinatal asphyxia
PICU: Pediatric intensive care unit
ROC: Receiver operating characteristics
ROI: Region of interest
SD: Standard deviation
TH: Therapeutic hypothermia
VABS: Vineland Adaptive Behavior Scales
