# Supplementary material for: The role of early functional neuroimaging in predicting neurodevelopmental outcomes in neonatal encephalopathy
Source: Eur J Pediatr. 2023 Jan 6;182(3):1191–200. doi: 10.1007/s00431-022-04778-0 (PMC10023620; doi:10.1007/s00431-022-04778-0)
Supplement: Supplementary file 1 — Supplementary file1 (DOCX 20 KB) [file 431_2022_4778_MOESM1_ESM.docx]

Functional stimulation paradigm

For visual stimulation [1], delivered on an LCD screen through a mirror fastened to the head coil, a checkerboard image was presented in 13 blocks of 20 seconds, flickering at frequencies of 1 to 4 hertz (Hz), interleaved with 14 baseline blocks (black screen) of 12 seconds. Auditory stimulus consisted of a 20-80 decibel (dB) tone, centered at 1.3 kilohertz (kHz) and frequency modulated over a range of ±1 kHz at a rate of 8 Hz, delivered through MRI compatible headphones incorporated with neonatal ear shields [2]. Sixteen blocks of 14 seconds were interleaved with 16 baseline blocks (no sound) of 14 seconds. For sensorimotor stimulation, bilateral forearm flexion/extension movements were induced by manual traction, at a frequency of ~1 Hz, by a physician positioned inside the scanner gantry [3].Ten blocks of motor stimulation of 14 seconds were interleaved with 11 baseline blocks (no movement) of 14 seconds.

1. Seghier ML, Lazeyras F, Huppi PS (2006) Functional MRI of the newborn. Semin Fetal Neonatal Med 11:479-488

2. Anderson AW, Marois R, Colson ER, Peterson BS, Duncan CC, Ehrenkranz RA, Schneider KC, Gore JC, Ment LR (2001) Neonatal auditory activation detected by functional magnetic resonance imaging. Magn Reson Imaging 19:1-5

3. Heep A, Scheef L, Jankowski J, Born M, Zimmermann N, Sival D, Bos A, Gieseke J, Bartmann P, Schild H, Boecker H (2009) Functional magnetic resonance imaging of the sensorimotor system in preterm infants. Pediatrics 123:294-300

**The role of early functional neuroimaging in predicting neurodevelopmental outcomes in neonatal encephalopathy**

European Journal of Pediatrics

Carla R Pinto^1^, João V Duarte, Carla Marques, Inês N Vicente, Catarina Paiva, João Éloi, Daniela J Pereira, Bárbara R Correia, Miguel Castelo-Branco, Guiomar Oliveira

^1^ Pediatric Intensive Care Unit, Hospital Pediátrico, Centro Hospitalar e Universitário de Coimbra, Coimbra, Portugal, Email: carla.regina.pinto@gmail.com; carla.pinto@chuc.min-saude.pt
