# Supplementary material for: The role of early functional neuroimaging in predicting neurodevelopmental outcomes in neonatal encephalopathy
Source: Eur J Pediatr. 2023 Jan 6;182(3):1191–200. doi: 10.1007/s00431-022-04778-0 (PMC10023620; doi:10.1007/s00431-022-04778-0)
Supplement: Supplementary file 2 — Supplementary file2 (DOCX 17 KB) [file 431_2022_4778_MOESM2_ESM.docx]

Auditory assessment

The formal auditory assessment was performed by an otorhinolaryngologist trained in pediatrics, including clinical evaluation, otoacoustic emissions, age-appropriate acuity measurements, tympanometry, and brainstem auditory evoked potentials performed at a median age of 13.5 (IQR 12 to 16.5) months, to allow to differentiate between conductive and sensorineural hearing loss. Tympanometry was performed using the tympanometer - TympStar^Tm^ (Grason Stadler, USA). Brainstem auditory evoked potentials were conducted through the Audera^Tm^ device (Grason Stadler, USA). The protocol consisted in 2006 repetitions of 100µs click ipsilateral stimuli, in rarefaction, with intensities between 100 and 30dB nHL, at a repetition rate of 33.10 stimuli per second, with a 40 DB nHL masking level, delivered through in-ear headphones. A high filter: 30 Hz@ -6 dB 12 dB/oct RC and low filter: 1.5 KHz in linear phase >40 dB/oct were used. The infants were placed in the parent’s lap in a quiet and comfortable environment.

Hearing loss impairment was defined and classified by the International Bureau for Audiophonology, according to tone loss as: normal or subnormal (below 20 dB); mild (between 21 and 40 dB); moderate (between 41 and 70 dB); severe (between 71 and 90 dB) and profound (over 90 dB).

**The role of early functional neuroimaging in predicting neurodevelopmental outcomes in neonatal encephalopathy**

European Journal of Pediatrics

Carla R Pinto^1^, João V Duarte, Carla Marques, Inês N Vicente, Catarina Paiva, João Éloi, Daniela J Pereira, Bárbara R Correia, Miguel Castelo-Branco, Guiomar Oliveira

^1^ Pediatric Intensive Care Unit, Hospital Pediátrico, Centro Hospitalar e Universitário de Coimbra, Coimbra, Portugal, Email: carla.regina.pinto@gmail.com; carla.pinto@chuc.min-saude.pt
