# Supplementary material for: The role of early functional neuroimaging in predicting neurodevelopmental outcomes in neonatal encephalopathy
Source: Eur J Pediatr. 2023 Jan 6;182(3):1191–200. doi: 10.1007/s00431-022-04778-0 (PMC10023620; doi:10.1007/s00431-022-04778-0)
Supplement: Supplementary file 3 — Supplementary file3 (DOCX 19 KB) [file 431_2022_4778_MOESM3_ESM.docx]

Visual assessment

The formal visual assessment was performed by an ophthalmologist experienced in pediatrics, including age-appropriate acuity measurements and visual evoked potentials performed at a median age of 15 (IQR 13 to 17) months. The visual assessment comprised Lea symbols and stereoacuity tests. Cerebral visual impairment was defined according to Lehman [1]. We used the device UTAS Visual Electrodiagnostic Testing System with an UBA-4204 amplifier (LKC Technologies, USA) to perform the visual evoked potentials. The protocol consisted of a reversal pattern stimulus with five phases (32x32,8x8,16x16,64x64,128x128) of a black and white checkboard. Eighty reversion stimulations were acquired, with an analysis time of 300ms, filters from 1 to 100Hz, checks of 2Hz, and contrast at 100%. The latencies of N75 and P100 waves were measured in ms. Participants were seated in front of a screen, placed one meter away at eye level. Fixation was continuously monitored. The testing procedure was conducted in a monocular and dim environment, with constant luminance.

1. Lehman SS (2012) Cortical visual impairment in children: identification, evaluation and diagnosis. Curr Opin Ophthalmol 23:384-387

**The role of early functional neuroimaging in predicting neurodevelopmental outcomes in neonatal encephalopathy**

European Journal of Pediatrics

Carla R Pinto^1^, João V Duarte, Carla Marques, Inês N Vicente, Catarina Paiva, João Éloi, Daniela J Pereira, Bárbara R Correia, Miguel Castelo-Branco, Guiomar Oliveira

^1^ Pediatric Intensive Care Unit, Hospital Pediátrico, Centro Hospitalar e Universitário de Coimbra, Coimbra, Portugal, Email: carla.regina.pinto@gmail.com; carla.pinto@chuc.min-saude.pt
