# Supplementary material for: The role of early functional neuroimaging in predicting neurodevelopmental outcomes in neonatal encephalopathy
Source: Eur J Pediatr. 2023 Jan 6;182(3):1191–200. doi: 10.1007/s00431-022-04778-0 (PMC10023620; doi:10.1007/s00431-022-04778-0)
Supplement: Supplementary file 4 — Supplementary file4 (DOCX 23 KB) [file 431_2022_4778_MOESM4_ESM.docx]

fMRI data during visual, auditory, and sensorimotor (motor) stimulation per participant and brain hemisphere

|  | **BOLD MEAN (% SIGNAL CHANGE)** | | | | | | **BOLD SEM (% SIGNAL CHANGE)** | | | | | |  |  |
| --- | --- | --- | --- | --- | --- | --- | --- | --- | --- | --- | --- | --- | --- | --- |
|  | VISUAL | | AUDITORY | | MOTOR | | VISUAL | | AUDITORY | | MOTOR | |  | |
|  | Left | Right | Left | Right | Left | Right | Left | Right | Left | Right | Left | Right | **NOTES** |  |
| NE01 |  | 0.251 | -0.211 | -0.23 | 0.951 | 0.622 |  | 0.053 | 0.064 | 0.065 | 0.249 | 0.123 |  |  |
| **NE02** |  | -0.366 | -0.309 | -0.328 | 0.941 |  |  | 0.094 | 0.077 | 0.067 | 0.274 |  | Bilateral spastic CP. Cerebral visual impairment. Sensorineural hearing loss |  |
| NE03 |  | -0.345 |  |  |  | -0.395 |  | 0.090 |  |  |  | 0.096 |  |  |
| NE04 | 0.147 | -0.214 | -0.136 |  |  |  | 0.032 | 0.078 | 0.025 |  |  |  |  |  |
| NE05 |  | 0.161 | -0.134 |  |  | 0.302 |  | 0.039 | 0.030 |  |  | 0.059 |  |  |
| NE06 | 0.464 | 0.515 |  |  | -0.32 |  | 0.147 | 0.149 |  |  | 0.122 |  |  |  |
| NE07 | -0.384 | -0.401 | -0.202 |  |  |  | 0.119 | 0.136 | 0.029 |  |  |  |  |  |
| **NE08** |  | 0.327 | -0.233 | 0.166 |  |  |  | 0.111 | 0.071 | 0.046 |  |  | Unilateral spastic CP (right) |  |
| **NE09** | 0.176 | 0.155 |  |  |  | 0.244 | 0.063 | 0.053 |  |  |  | 0.034 | Bilateral spastic CP |  |
| NE10 | -0.236 |  |  | 0.252 |  | 0.77 | 0.071 |  |  | 0.041 |  | 0.136 |  |  |
| NE11 | -0.131 | 0.171 |  | 0.176 | 0.883 | 1.127 | 0.026 | 0.029 |  | 0.032 | 0.114 | 0.185 |  |  |
| NE12 | 0.135 |  | 0.22 | 0.159 | 1.459 | 2.385 | 0.038 |  | 0.058 | 0.029 | 0.174 | 0.329 |  |  |
| NE13 |  |  | -0.154 | -0.161 |  | 1.436 |  |  | 0.044 | 0.028 |  | 0.249 |  |  |
| NE14 | 0.137 |  | 0.166 | 0.14 | 0.884 | 0.716 | 0.032 |  | 0.039 | 0.037 | 0.137 | 0.087 |  |  |
| NE15 |  | -0.191 | -0.237 | 0.193 |  | 0.207 |  | 0.034 | 0052 | 0.040 |  | 0.053 | Left brachial plexus injury |  |
| NE16 | -0.176 | 0.131 |  |  |  | 0.649 | 0.040 | 0.034 |  |  |  | 0.104 |  |  |
| NE17 |  |  | -0.381 | -0.333 | -0.867 |  |  | 0.053 | 0.124 | 0.102 | 0.201 |  |  |  |
| **NE18** | -0.14 |  |  | -0.169 |  | -0.499 | 0.029 | 0.094 | 0.064 | 0.027 |  | 0.092 | Bilateral spastic CP. Cerebral visual impairment |  |

Responses are reported as mean, standard error of the mean (SEM) of the % of BOLD signal change during periods of stimulation relative to baseline. Empty cells are due to non-significant signal change in expected regions of interest, respective to the type of stimulation. Participants with ID in bold lettering are those with severe disability. fMRI - functional magnetic resonance imaging; BOLD - blood-level-oxygen-dependent; NE - neonatal encephalopathy; CP - cerebral palsy

**The role of early functional neuroimaging in predicting neurodevelopmental outcomes in neonatal encephalopathy**

European Journal of Pediatrics

Carla R Pinto^1^, João V Duarte, Carla Marques, Inês N Vicente, Catarina Paiva, João Éloi, Daniela J Pereira, Bárbara R Correia, Miguel Castelo-Branco, Guiomar Oliveira

^1^ Pediatric Intensive Care Unit, Hospital Pediátrico, Centro Hospitalar e Universitário de Coimbra, Coimbra, Portugal, Email: carla.regina.pinto@gmail.com; carla.pinto@chuc.min-saude.pt
