# Supplementary material for: The role of early functional neuroimaging in predicting neurodevelopmental outcomes in neonatal encephalopathy
Source: Eur J Pediatr. 2023 Jan 6;182(3):1191–200. doi: 10.1007/s00431-022-04778-0 (PMC10023620; doi:10.1007/s00431-022-04778-0)
Supplement: Supplementary file 5 — Supplementary file5 (DOCX 18 KB) [file 431_2022_4778_MOESM5_ESM.docx]

fMRI BOLD responses by type of sedation in newborns with neonatal encephalopathy

| **fMRI BOLD responses** (% signal change) per stimulus and hemisphere | **Sedation Type** | | **p** |
| --- | --- | --- | --- |
|  | **Midazolam**  (n=7) | **Propofol**  (n=11) |  |
| **Visual left**, mean ±SD | 0.757 ±0.428 | -0.0336 ±0.175 | 0.563 |
| **Visual right**, mean ±SD | -0.057 ±0.363 | -0.119 ±0.189 | 0.303 |
| **Auditory left**, median (IQR) | -0.202 (-0.26 to 0.135) | -0.193 (-0.273 to 0.195) | 1.0 |
| **Auditory right**, median (IQR) | -0.279 (-0.328 to -0.279) | 0.159 (-0.165 to 0.184) | 0.145 |
| **Sensorimotor left**, mean ±SD | 0.524 ±0.731 | 0.52±1.001 | 0.928 |
| **Sensorimotor right**, mean ±SD | 0.176 ±0.52 | 0.782 ±0.821 | 0.266 |

fMRI - functional magnetic resonance imaging; BOLD - blood level oxygen dependent; n - number; SD - standard deviation; IQR - interquartile range

**The role of early functional neuroimaging in predicting neurodevelopmental outcomes in neonatal encephalopathy**

European Journal of Pediatrics

Carla R Pinto^1^, João V Duarte, Carla Marques, Inês N Vicente, Catarina Paiva, João Éloi, Daniela J Pereira, Bárbara R Correia, Miguel Castelo-Branco, Guiomar Oliveira

^1^ Pediatric Intensive Care Unit, Hospital Pediátrico, Centro Hospitalar e Universitário de Coimbra, Coimbra, Portugal, Email: carla.regina.pinto@gmail.com; carla.pinto@chuc.min-saude.pt
