# Supplementary material for: The role of early functional neuroimaging in predicting neurodevelopmental outcomes in neonatal encephalopathy
Source: Eur J Pediatr. 2023 Jan 6;182(3):1191–200. doi: 10.1007/s00431-022-04778-0 (PMC10023620; doi:10.1007/s00431-022-04778-0)
Supplement: Supplementary file 6 — Supplementary file6 (DOCX 19 KB) [file 431_2022_4778_MOESM6_ESM.docx]

Griffiths Mental Development Subscales and Vineland Adaptive Behaviour Subdomains at 18 months

| GMDS Subscales, DQ, N | NE  (n=18) | VABS Subdomains  DQ, N | NE  (n=18) |
| --- | --- | --- | --- |
| Locomotor  <70  70-84  ≥85 | 4  0  14 | **Communication**  <70  70-84  ≥85 | 1  12  5 |
| Personal-Social  <70  70-84  ≥85 | 3  0  15 | **Socialization**  <70  70-84  ≥85 | 2  2  14 |
| Hearing and Language  <70  70-84  ≥85 | 2  1  15 | **Daily Living**  <70  70-84  ≥85 | 3  9  6 |
| Eye and Hand Coordination  <70  70-84  ≥85 | 4  0  14 | **Motor Skills**  <70  70-84  ≥85 | 4  0  14 |
| Performance  <70  70-84  ≥85 | 4  0  14 |  |  |
| Practical Reasoning^a^  <70  70-84  ≥85 | 0  0  1 |  |  |

GMDS - Griffiths Mental Development Scales; VABS - Vineland Adaptive Behavior Scales; DQ - development quotient; n - number; NE - neonatal encephalopathy

^a^ Only one child was old enough to perform Practical Reasoning subscale

**The role of early functional neuroimaging in predicting neurodevelopmental outcomes in neonatal encephalopathy**

European Journal of Pediatrics

Carla R Pinto^1^, João V Duarte, Carla Marques, Inês N Vicente, Catarina Paiva, João Éloi, Daniela J Pereira, Bárbara R Correia, Miguel Castelo-Branco, Guiomar Oliveira

^1^ Pediatric Intensive Care Unit, Hospital Pediátrico, Centro Hospitalar e Universitário de Coimbra, Coimbra, Portugal, Email: carla.regina.pinto@gmail.com; carla.pinto@chuc.min-saude.pt
