# Supplementary material for: The role of early functional neuroimaging in predicting neurodevelopmental outcomes in neonatal encephalopathy
Source: Eur J Pediatr. 2023 Jan 6;182(3):1191–200. doi: 10.1007/s00431-022-04778-0 (PMC10023620; doi:10.1007/s00431-022-04778-0)
Supplement: Supplementary file 7 — Supplementary file7 (DOCX 18 KB) [file 431_2022_4778_MOESM7_ESM.docx]

Accuracy of the fMRI measures to predict the absence of sensorineural hearing loss, cerebral visual impairment, and cerebral palsy

| **fMRI measures (BOLD mean - % signal change)**  **during auditory stimulation in sensorineural hearing loss** | |
| --- | --- |
| **Brain hemisphere** | **AUC, [95% CI]** |
| Left | 0.9 [0.714;1.0] |
| Right | 0.9 [0.714;1.0] |
| **fMRI measures (BOLD mean - % signal change)**  **during visual stimulation in cerebral visual impairment** | |
| **Brain hemisphere** | **AUC, [95% CI]** |
| Left | 0.667 [0.359;0.975] |
| Right | 0.909 [0.739;1.0] |
| **fMRI measures (BOLD mean - % signal change)**  **during sensorimotor stimulation in cerebral palsy** | |
| **Brain hemisphere** | **AUC, [95% CI]** |
| Left | 0.333 [0.0;.711] |
| Right | 0.9 [0.694;1.0] |

AUC - area under the curve; CI - confidence intervals; fMRI - functional magnetic resonance imaging; BOLD - blood-oxygen-level-dependent

**The role of early functional neuroimaging in predicting neurodevelopmental outcomes in neonatal encephalopathy**

European Journal of Pediatrics

Carla R Pinto^1^, João V Duarte, Carla Marques, Inês N Vicente, Catarina Paiva, João Éloi, Daniela J Pereira, Bárbara R Correia, Miguel Castelo-Branco, Guiomar Oliveira

^1^ Pediatric Intensive Care Unit, Hospital Pediátrico, Centro Hospitalar e Universitário de Coimbra, Coimbra, Portugal, Email: carla.regina.pinto@gmail.com; carla.pinto@chuc.min-saude.pt
